# An Overview on Atopic Dermatitis, Oxidative Stress, and Psychological Stress: Possible Role of Nutraceuticals as an Additional Therapeutic Strategy

**DOI:** 10.3390/ijms25095020

**Published:** 2024-05-04

**Authors:** Clara Alessandrello, Serena Sanfilippo, Paola L. Minciullo, Sebastiano Gangemi

**Affiliations:** School and Operative Unit of Allergy and Clinical Immunology, University Hospital of Messina, 98125 Messina, Italy; clara.alessandrello@outlook.it (C.A.); serenasanfilippo.doc@gmail.com (S.S.); sebastiano.gangemi@unime.it (S.G.)

**Keywords:** atopic dermatitis, oxidative stress, psychological disorders, alexithymia, depression, anxiety, nutraceuticals, natural compounds

## Abstract

Atopic dermatitis (AD) is a chronic inflammatory skin condition with a considerable impact on patients’ quality of life. Its etiology is multifactorial and, among the predisposing factors, a role is played by oxidative stress. Pollution, recurrent infections, and psychological stress contribute to oxidative stress, amplifying the production of proinflammatory cytokines and worsening barrier damage. There are various oxidative stress mechanisms involved in the pathogenesis of AD. Moreover, AD often appears to be associated with psychological disorders such as alexithymia, depression, and anxiety due to severe itching and related insomnia, as well as social distress and isolation. The increasing incidence of AD requires the evaluation of additional therapeutic approaches in order to reduce the psychological burden of this condition. Our review aims to evaluate the role of some nutraceuticals in AD treatment and its related psychological comorbidities. The combination of some natural compounds (flavonoids, alkaloids, terpenes, isothiocyanates) with traditional AD treatments might be useful in improving the effectiveness of therapy, by reducing chronic inflammation and preventing flare-ups, and in promoting corticosteroid sparing. In addition, some of these nutraceuticals also appear to have a role in the treatment of psychological disorders, although the underlying oxidative stress mechanisms are different from those already known for AD.

## 1. Introduction

Atopic dermatitis (AD), also known as atopic eczema, is a chronic inflammatory skin disease characterized by skin xerosis, intense itching, and susceptibility to cutaneous infections, affecting children at all ages but also adults, with phases of relapse and remission. It is a common condition worldwide that has a significant impact on patients’ quality of life [1].

Depending on the correlation with the total IgE value, it can be classified into the following: an extrinsic or atopic form and an intrinsic or non-atopic form. AD is often the first step of “atopic march”, so it is frequently associated with allergic rhinitis, asthma, and food allergy [2].

Interestingly, in children with AD who progress to atopic march, an increased risk of neurodevelopmental diseases [3,4] and other nonallergic comorbidities (metabolic diseases, obesity, cardiovascular, and specific cancers) has been observed [5].

AD has a complex and multifactorial pathogenesis: genetic predisposition to skin barrier alterations (filaggrin [FLG], loricrin [LOR], and involucrin [IVL] gene mutations), an alteration of lipid metabolism resulting in an increase in transepidermal water loss (TEWL) (reduction in ceramides), and innate or adaptive immune dysregulation (keratinocytes, innate lymphoid cells, T helper lymphocytes) [6].

AD is traditionally characterized by biphasic inflammation: Th2-inflammation is predominant in the acute phase, with the production of IL-4, IL-5, IL-13, TSLP, IL-25, and IL-31; next, a Th2-Th1 switch occurs, leading to a chronic phase (IFN-γ, IL-12, IL-5, and GM-CSF). In addition, IL-9, IL-17, and IL-22 are also produced, with the roles of Th9, Th17, and Th22 underlying the induction and maintenance of AD [7,8].

The overexpression of Th2 and Th22 cytokines contributes to skin barrier damage. FLG is a pivotal barrier protein and its alterations modify corneocytes and reduce the inflammatory threshold toward irritants and haptens. FLG is decreased in the majority of patients with AD, predisposing to early onset or persistent forms. Keratinocytes also produce antimicrobial peptides (AMPs), such as cathelicidin (LL-37) and beta-defensins (HBDs), but their expression is inhibited by Th2 cytokines; for this reason, in AD, there is a greater propensity for Staphylococcus aureus infections [9,10]. According to a 2019 meta-analysis, a possible association between AD risk and particular TLR2 and TLR4 polymorphisms exists [11]. In AD, alterations of enzymes involved in the production of free fatty acids and ceramides change the intercellular lipid composition of the stratum corneum, contributing to TEWL and the resulting skin xerosis and itching [12].

Environmental pollution appears to play an important role in the pathogenesis of AD: volatile organic compounds, traffic-related air pollutants, and second-hand smoke exposure seem to increase the incidence of allergic diseases, including AD. Environmental pollutants cause oxidative stress, by the alteration of cutaneous proteins and lipids, so they worsen inflammation and destroy the skin barrier [13,14,15]. In addition, it has been observed that itching and scratching worsen oxidative stress in mouse models [16].

In this review, the involvement of oxidative stress in AD pathogenesis and the psychological comorbidities of AD patients are reported with an overview of the role of some natural products that might be used to manage this complex disease.

The search was conducted in PubMed via the terms “atopic dermatitis” AND “anxiety”, “atopic dermatitis” AND “depressive disorder”, “atopic dermatitis” AND “alexithymia”, and “atopic dermatitis” AND “oxidative stress”. Only articles published in English were considered. The articles were then sorted by relevance and screened for consistency with the aim of the review, also selecting articles that included the effects of nutraceuticals on atopic dermatitis and mood disorders.

## 2. Role of Oxidative Stress in AD

Oxidative stress results from an imbalance between oxidant and antioxidant factors and mechanisms. The prevalence of oxidants results in tissue damage and chronic inflammation that underlies many inflammation-based diseases, such as sepsis, acute lung injury, the ischemia–reperfusion state, and atherosclerosis [17].

Inflammation in AD is Th2-mediated in the initial phase, and, together with skin barrier disruption and barrier protein deficits, will lead to the chronic phase of inflammation, characterized by the release of pro-inflammatory cytokines such as IL-1 and TNF-α. From a histopathological point of view, skin inflammation in AD results in a dense infiltrate of T lymphocytes, monocyte–macrophages, and eosinophils, which release a large number of cytokines and chemokines [18].

A proinflammatory cytokine environment can induce keratinocytes, Langerhans cells, and dermal fibroblasts to overproduce reactive oxygen species (ROS), such as superoxide anions (O_2_^2−^) and hydrogen peroxide (H_2_O_2_) [19], and nitric oxide (NO) via the action of the inducible nitric oxide synthase (iNOS). Elevated levels of iNOS have been found in the dermal microvasculature of AD patients [20]. In turn, oxidative stress products amplify the inflammatory response through the upregulation of genes coding for proinflammatory cytokines [21].

In addition, other factors contribute to oxidative stress in AD, such as pollution, psychological stress, and infection [22]. Staphylococcus aureus infections are very common in patients with AD due to intrinsic skin barrier defects and contribute to acute exacerbations, also via increased oxidative stress. During infection, activated neutrophils, via the Myeloperoxidase (MPO) system, produce hypochlorite (HOCl), which oxidizes free amino acids with the formation of Advanced glycation end products (AGEs). AGEs contribute to increased endothelial permeability and activate macrophages and endothelial cells, accelerating inflammation and oxidative stress [18].

Therefore, oxidative stress appears to play a nontrivial role in the genesis of AD, in close association with the inflammatory mechanisms of Th2 and Th1 immunity. Proteins are extremely sensitive to increased oxidative stress, as ROS react with amino acid residues, changing their structure and resulting in a loss of biological activity. Indeed, elevated serum levels of Advanced Oxidation Protein Products (AOPPs) have been found in patients with AD compared with healthy controls [23]. Serum thiols together with glutathione and its antioxidant enzymes are responsible for the scavenging of free radicals. Thiol (SH) levels were significantly lower in patients with AD and, at the same time, disulfide (SS) levels were significantly higher (higher ratio of SS/SH). The impairment of this SH-SS balance and its shift toward increased oxidation would seem to be responsible in children with AD for the cellular damage of keratinocytes, which are sensitive to lipid peroxidation [24]. Indeed, in AD, oxidative stress occurs predominantly in plasma lipids with HDL peroxidation and the production of lipid hydroperoxides. MPO, released into the circulation by activated leukocytes, could be the enzyme responsible for HDL peroxidation and its functional inactivation. On the other hand, paraoxonases (PONs) and particularly paraoxonase 1 (PON1) play an antioxidant role by modulating the susceptibility of serum HDL to lipid peroxidation [22]. In fact, high serum levels of MPO and low levels of PON1 have been found in patients with AD, further demonstrating the imbalance between oxidant and protective antioxidant factors [25]. In addition, thiols act on the regulation of skin inflammation in a dose-dependent manner, reducing the polarization of Th2 lymphocytes and the production of IgE, IgG4, IL-4, IL-5, and IL-13 [26].

Biomarkers of oxidative stress have also been studied at the urinary level in patients with AD. This turns out to be a noninvasive method and, therefore, ideal for assessing oxidative stress levels. In particular, urinary nitrate levels, including 8-Hydroxy-2’-deoxyguanosine (8-OHdG) and malondialdehyde (MDA), have been positively correlated with the degree of severity of AD [27]. In addition, high urinary levels of 8-OHdG in children with AD would appear to be predictive of the advancement of atopic march, with an increased risk of asthma onset [28]. Finally, it would appear that 8-OHdG levels fluctuate according to the phases of disease activity, increasing in the flare-up phases and decreasing in the remission phases [29].

There is also an antioxidant system that counteracts the effect of oxidative stress to maintain skin homeostasis. The main enzymes involved are superoxide dismutase (SOD), glutathione peroxidase (GPx), and catalase (CAT). SOD is a mitochondrial matrix enzyme that helps to eliminate O_2_^2−^, reducing the damage created by lipid peroxidation, DNA damage, and collagen degradation. GPx is an enzyme found in both mitochondrial and cytosolic levels, and it cooperates with SOD in the destruction of H_2_O_2_ and lipid hydroperoxides, reducing inflammation and promoting healing of injured skin. The activity of GPx is closely connected to the GSH/GSSG cycle through glutathione reductase. CAT is located in peroxisomes and also promotes the degradation of H_2_O_2_, but it has a much more powerful action than GPx. There are also nonenzymatic antioxidants (carotenoids, tocopherol, and ascorbic acid) that have a high capacity for quenching singlet oxygen and trapping peroxyl radicals. Increased lipid peroxidation in AD seems to reduce SOD, CAT, and GPx activities and decrease levels of nonenzymatic antioxidants [30].

In children with AD and elevated levels of oxidant factors, a compensatory mechanism to counteract the action of oxidant products could be melatonin production. The mean serum melatonin levels in these patients were found to be higher, which could contribute to the reduction in skin inflammation [31]. Melatonin levels have also been studied in correlation with MDA. High levels of the MDA/melatonin ratio have been correlated with more severe forms of AD [32]. Another antioxidant marker studied is tocopherol. Tocopherol predominates at the scalp level and is contained in many topical products used for AD. One study has demonstrated levels of tocopherol in AD patients two times higher than those in healthy patients, which could not be correlated purely with the use of creams containing tocopherol. Endogenous tocopherol probably increases in response to the chronic skin inflammation, to counterbalance excessive oxidative stress [33].

The role of oxidative stress in the development of AD is of great interest and is also being studied in the prenatal stages of life. There may be a link between psychophysical stress and oxidative stress. One study has looked at the relationship between maternal stress and oxidative stress levels in a child with AD. It appears that levels of oxidative stress in the child may be dependent on maternal stress. Chang et al. observed that children of stressed mothers had higher levels of glucocorticoids, which activate ROS and downregulate antioxidant systems such as glutathione/GSSG (oxidized glutathione). In addition, elevated levels of cortisol persistently activate the hypothalamic–pituitary–adrenal axis, which modulates immune responses and may contribute to the increased susceptibility of these children to developing AD [34].

Table 1 summarizes the main oxidative stress markers involved in the pathogenesis of AD and the associated antioxidant factors that should regulate the oxidation/antioxidation balance.

**Figure 1:**
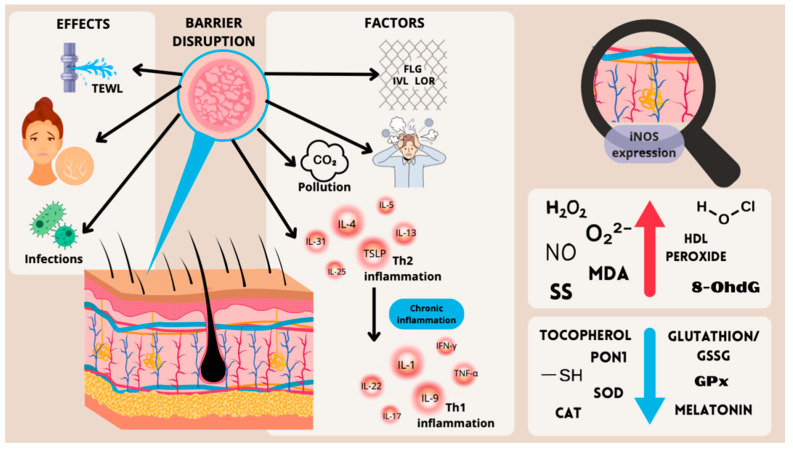
Inflammatory and oxidative factors contributing to the pathogenesis of atopic dermatitis (TEWL = trans-epithelial water loss; FLG = filaggrin; IVL = involucrin; LOR = loricrin; iNOS = inducible nitric oxide synthase; H_2_O_2_ = hydrogen peroxide; O_2_^2−^ = superoxide; NO = nitric oxide; MDA = malondialdehyde; SS = disulfide; HOCl = hypochlorite; 8-OHdG = 8-Hydroxy-2′-deoxyguanosine; PON1 = paraoxonase 1; SH = thiol; SOD = superoxide dismutase; CAT = catalase; GSSG = oxidized glutathione; GPx = glutathione peroxidase).

Figure 1 illustrates the main pathogenetic mechanisms underlying the manifestations of AD, including the effects of chronic inflammation and oxidative stress.

## 3. Psychological Stress Associated with AD

The psychological sphere appears to play a contributory role in many chronic diseases, such as cancer, and in dermatoses such as AD, urticaria, and psoriasis. The mechanisms linking psychological stress and disease are still not very clear. Some studies have found altered humoral and cellular immunity during psychological stress. In other studies, sustained psychological stress has been correlated with delayed wound healing and with skin barrier defects and TEWL in AD [35].

AD is a chronic relapsing dermatosis, and flare-ups often coincide with periods of severe psychological and physical stress. Patients with AD would appear to have a characteristic psychological profile: they are aggressive and depressed, with high levels of anxiety and sensitivity; and they often they suffer from insomnia, which contributes to the psychological state. Anxiety and depression have been correlated with the severity of skin lesions and the degree of itching [36]. In addition, in patients with manifesting skin diseases, psychological stress may also result from embarrassment, lack of self-confidence, and the non-acceptance of one’s physical appearance and pathology [37]. Therefore, it is possible to refer to an actual comorbidity between psychiatric conditions, such as depression, anxiety, and alexithymia, and skin conditions, such as AD.

### 3.1. AD and Alexithymia

Alexithymia is the inability to identify, recognize, and understand one’s own emotions, associated with a difficulty in describing feelings to other people and a lack of imaginative processes. The term alexithymia was introduced by Sifneos and it is derived from the Greek “alexis” (no words) and “thymos” (emotion) [38]. The most used questionnaire for alexithymia is the revised 20-item version Toronto Alexithymic Scale (TAS-20) [39]. It has a prevalence of 10% in the general population and it is associated with anxiety and/or depressive disorders, autism spectrum disorder, addictive disorder, and eating disorders [40]. High alexithymic scores are present in patients with myocardial infarction, inflammatory bowel disease, and recurrent severe asthma [41,42]. Preliminary data show that alexithymia seems to be associated with AD, alopecia areata, psoriasis, vitiligo, and chronic urticaria. Probably, it seems to be associated with changes in immunity, sympathetic activity, and brain activity; some researchers correlate alexithymia with early traumatic experiences and insecure attachment with caregivers [43,44,45]. A case–control study by Poot et al. evaluated the family dysfunction of patients with psoriasis, AD, and alopecia areata, and patients with minor skin condition control. Overall, the prevalence of anxiety and/or depression was 43.3%, particularly 71.4% in patients with AD. The prevalence of alexithymia was 66.7% in patients with AD and psoriasis; in addition, the correlation between TAS-20 score and Genogram score was significant only in AD patients. Family dysfunction seems to be a keystone in the onset of exacerbation of AD [46].

A cross-sectional study by Chiricozzi et al. investigated the occurrence of alexithymia in AD patients, compared to healthy controls (HCs). They showed an alexithymic trait (TAS-20 ≥ 51) in 62.4% of AD patients (vs. 29.2% in HC), alexithymia (TAS-20 ≥ 61) in 27.7% of AD patients (vs. 7.5% in HC), and borderline alexithymia in 34.6% of AD patients (vs. 21.7% in HC). Furthermore, they also observed that alexithymia was more common in patients with severe AD (correlating with itching intensity and sleep disturbances) and the EASI score value ≥21 could be a predictive factor [47].

### 3.2. AD and Anxiety/Depressive Disorders

Anxiety disorders (generalized anxiety disorder, agoraphobia, panic disorder, social anxiety disorder, specific phobias, separation anxiety disorder, and selective mutism) are one of the most common mental disorders with a high prevalence worldwide. Anxiety is characterized by an excessive and persistent fear and worry, and an avoidance of public events, to the point of impairing the person’s social or occupational functioning. It often coexists with other psychiatric disorders, such as depression and substance abuse [48,49].

Depressive disorders (major depressive disorder, dysthymic disorder, subthreshold depression, melancholia, anxious distress, and other minor depressive disorders) have a high prevalence in the world population, with a higher frequency in women and an onset between adolescence and 40 years of age. Depression is characterized by anhedonia, reduced motivation, and a disruption of daily activities; it is often misdiagnosed and associated with serious consequences like suicide. The two most commonly used classificatory diagnostic systems are the Diagnostic and Statistical Manual of Mental Disorders (DSM-5) and International Classification of Diseases (ICD-10) [50,51].

Depression appears to be correlated to some stress-related chronic conditions, due to subsequent prolonged activation of the hypothalamic–pituitary–adrenal axis [52,53]; furthermore, both metabolic and immunological dysregulations are also associated with depression [54].

Since 1994, a statistically significant correlation was observed between chronic inflammatory skin conditions (psoriasis, AD, chronic spontaneous urticaria) and depression [55]. A multicenter observational cross-sectional study confirmed that the association of depression and anxiety was highest in patients with chronic skin diseases, such as AD [56]. Moreover, another study in 2019 observed that AD is associated with mental disorders (especially depressive symptoms) much more than other chronic skin conditions [57]. The same association is noted in anxiety disorders [58].

Several studies have found that AD is a shared risk factor for both depression and/or anxiety [59], although the topic is controversial [60,61,62,63,64,65,66]. By administering the State/Trait Anxiety Inventory to a large sample of French allergic patients (affected by asthma, allergic rhinitis, AD, food allergy, urticaria, etc.), it was observed that high mean scores for state and trait anxiety were mostly associated with AD [67]. Looking at the National Eczema Society of United Kingdom, about 46% of AD patients were shown to have anxiety and depression [68]. According to these studies, Cheng et al. observed that AD patients without psychiatric history have an increased risk of developing major depression, any depressive disorder, and anxiety disorders [69]. A cross-sectional study by Yang et al. showed that both atopic and non-atopic eczema were significantly associated with an increased likelihood of depressive disorders; nevertheless, the association of depression with AD is not due to total IgE levels and thus to IgE-mediated mechanisms [70].

Furthermore, Wei et al. investigated the temporal association between atopic diseases (asthma, allergic rhinitis, AD, or allergic conjunctivitis) and mood disorders (major depression and bipolar disorder) in a cohort of atopic adolescents, who had no psychiatric history, compared to a non-atopic cohort. This study showed a higher incidence of major depression, any depressive disorder, and bipolar disorder in the atopic cohort, with an earlier onset of symptoms [71]. In addition, it was observed that the Beck Depression Inventory and Self-rating Anxiety Scale questionnaires were higher in patients with severe AD [36], in agreement with Salfi et al., who found that more severe forms of AD were associated with more severe symptoms of depression and anxiety [72].

Hence, special attention should be paid by clinicians to the mental health status of patients with AD. This could give clinicians one more tool to prevent exacerbations of mental illnesses [73] so that the therapeutic response can also be improved by psychotherapy [74,75].

Since there seems to be a probable correlation between AD and anxiety-depressive disorders, some authors have tried to assess the probable underlying causes. In 2001, an observational study provided the first link between psychological state and skin function in humans. A group of healthy university students were assessed at three different times—during university examinations, presumed as higher-stress, and during winter and spring vacations, presumed as lower-stress—evaluating the level of psychological stress and barrier integrity. In contrast to basal TEWL levels, repeated-measures analysis revealed significant changes in the rates of barrier recovery across the three periods, which corresponded in parallel with an increase in perceived psychological stress during the higher- versus the initial lower-stress occasions [35].

It is known that the immune system and brain communicate with each other. Glucocorticoids and catecholamines (stress hormones) cause Th1 suppression, by the production of IL-10 and inhibition of IL-12. In addition, proinflammatory cytokines cause a dysfunction of glucocorticoid receptors, usually present in depression disorders. IL-4, the main Th2-driver, appears to play a role in serotonin metabolism underlying depressive disorders (especially with suicidal tendencies). Furthermore, it has been observed that proinflammatory cytokines cause a reduction in tryptophan, the precursor of serotonin [76]. Kimata dosed neurotrophins (NGF, NT-3, NT-4, and BDNF) in cerebrospinal fluid (CSF), observing no difference between healthy controls and AD patients without suicide attempts. NGF and NT-3 were significantly higher and NT-4 and BDNF were lower in AD patients with suicide attempts. No significant correlation was found according to the degree of AD severity. A change in CSF levels of neurotrophins may be due to depression or anxiety and AD could contribute to this, acting as a potential marker to be studied [77,78]. It has been observed that in patients who received treatment with tandospirone citrate (TC, a serotonin agonist for the 5-HT1A receptor), POMS scores for tension–anxiety and the SCORAD Index decreased significantly; no significant change in the untreated patients was reported. In murine models, different types of stress induce the release of neuropeptides and neurotransmitters (including substance P, neurotensin, calcitonin-gene-related peptide, and CRH). TC may have a direct agonistic effect on receptors on dermal mast cells and other inflammatory cells, preventing their degranulation [79].

Another study found that postpartum depression enhances the risk of childhood AD even when other social demographic and maternal mental health indexes are considered. The effect could be due to either the rise in cortisol levels that can disrupt the skin’s barrier function or epigenetic regulation by DNA methylation or histone deacetylation of the glucocorticoid receptor gene [80,81].

Another hypothesis about the correlation between AD and anxiety/depression is closely related to altered sleep quality due to itching [82]. Zachariae et al. confirmed that pruritus is the better predictor of anxiety, depression, and quality of life impairment. The association between psychological and somatic symptoms is partly mediated by the effect of pruritus on sleep quality, to such an extent that adjusting the data for sleep impairment, this association ceased to be statistically significant [83]. A recent cross-sectional study observed that more severe AD symptoms were associated with more severe symptoms of insomnia, anxiety, and depression; moreover, the severity of insomnia was correlated with symptoms of anxiety and depression. Thus, insomnia could explain the relationship between AD severity and anxiety/depressive symptoms [72].

In summary, at this time, the correlation between AD and psychological disorders seems certain. However, it is not easy to determine only through questionnaires the presence or absence of psychological disorders in the early life (the period when AD predominates). Therefore, it is difficult to understand whether AD and psychological disorders share some molecular mechanisms or whether the development of anxiety and depression at a young age is a consequence of the distress experienced by AD patients. Further studies are needed to better understand the causation of this association.

## 4. Natural Compounds as an Alternative Treatment for AD

The incidence of AD is increasing day by day, making it necessary to find new therapeutic strategies for this disease, which also impacts patients’ psychological health in order to improve their quality of life.

The treatment of AD relies mainly on topical drugs, such as corticosteroids, calcineurin inhibitors, and emollient creams to counteract TEWL and skin dryness [84]. Topical products have good penetration ability at the skin level, although in the chronic stages of the disease, they often fail to reach the deeper layers of the skin [85].

In severe flare-ups, however, patients are often forced to use systemic medications such as corticosteroids and antibiotics in the case of bacterial overinfection [86].

In the more severe and difficult-to-treat forms of AD, biologic drugs, such as dupilumab or Janus kinase inhibitors, have come to the rescue [87,88,89].

All of these factors contribute to patients’ great discomfort in daily life because of their physical appearance, and this contributes to the stress they are subjected to, which can also worsen the course of the disease and the flare-ups of lesions [90].

Therefore, it might be useful to combine these conventional treatments with the use of natural products to enhance the effectiveness of therapy, in terms of reducing chronic inflammation and oxidative stress mechanisms, improving the antioxidant system’s functioning, in order to reduce flare-ups and improve skin appearance [91].

Natural compounds contained in the foods we consume daily may not only play an adjuvant and synergistic action with traditional therapies for diseases such as AD but could also have a beneficial effect on comorbidities that may be associated with AD, such as psychological disorders [92]. Pathologies such as anxiety, depression, and alexithymia are frequently found in patients with AD, and some pathogenic pathways could probably be common between AD and these psychological conditions, related to the chronic inflammatory state and dysregulation of the oxidative and antioxidant mechanisms [93].

Below, we describe the characteristics of the well-characterized natural compounds that have demonstrated beneficial, anti-inflammatory, and antioxidant action at the level of AD lesions.

### 4.1. Flavonoids

Flavonoids are a group of pigments contained in plants with a wide range of biological actions. These compounds are largely responsible for the colors of plants, flowers, and fruits, particularly the brilliant shades of blue, scarlet red, and orange [94]. In addition to various types of vegetables and fruits (especially in citrus fruits, apples, and grapes), flavonoids are also found in seeds, nuts, cereals, spices, herbs (rosemary, thyme, and parsley), legumes, onions, and various medicinal plants; in addition, various beverages such as wine (especially red wine) and black and green tea contain appreciable amounts of flavonoids [95].

*Wheatgrass* is known for its high flavonoid and polyphenol content and is therefore used in the form of extracts, powders, and juices as a dietary supplement [96]. *Aronia melanocarpa* is a fruit belonging to the Rosaceae family, consumed in the form of juices, jams, and fruits that contain various bioactive compounds, such as anthocyanins, polyphenols, and flavonoids [97]. The extract of the above two natural compounds (TAAR) has been orally administered to AD mice to treat skin lesions, and *chlorogenic acid*, a well-known dietary polyphenol, could be the major factor responsible for the improvement in AD symptoms. It dose-dependently reduces the mRNA expression of iNOS, cyclooxygenase 2 (COX2), NO, and prostaglandin E2 (PGE2), and on the other hand, chlorogenic acid results in the increased expression of antioxidant protective factors, such as nuclear factor erythroid 2-related factor 2 (Nrf2) and heme oxygenase-1 (HO-1). Moreover, the antioxidant effect obtained in TAAR-treated patients is not obtained in dexamethasone-treated patients [98].

*Nymphoides peltata* (NP) is an edible aquatic plant in the Menyanthacea family, found in central Europe and the temperate and subtropical regions of Eurasia. The extract of Nymphoides species has bioactive properties with anti-inflammatory and antioxidant actions and contains compounds such as flavonoids, phenolic acid, tannins, saponins, triterpenoids, ephedrine, ephedradin C, 4-hydroxycoumarin, and benzopyrone. The topical application of NP extract increases the expression of the antioxidant enzymes Nrf2 and HO-1, which regulate excessive oxidative stress in the skin affected by AD, and it would again appear that chlorogenic acid, together with 3,5-dicaffeoylquinic acid and 3,4-dicaffeoylquinic acid, improves AD-related inflammation and oxidative stress by activating the Nrf2/HO-1 defense pathway [99].

The *chlorogenic acid* and *caffeic acid* present in *Coffea arabica* extract (CAE) have been shown to have antioxidant activity both in vitro and in vivo. In vitro CAE treatment results in reduced levels of IFN- γ/TNF-α-induced intracellular ROS and increased levels of antioxidant enzymes such as CAT, thereby improving the balance between oxidant and antioxidant factors at the skin level [100]. In vivo, caffeic acid not only improves AD lesions, by reducing TEWL and restoring the skin barrier and IVL, FLG, and LOR, but also gives the best results in terms of scavenging free radicals and inhibiting lipid peroxidation [101].

*Blueberries* and *black rice* are sources of antioxidants belonging to the flavonoid family, i.e., anthocyanins and derivatives of gallic acid, whose anti-inflammatory and antioxidant activity is enhanced in Lactobacillus plantarum-supplemented and -fermented (FBBBR) forms. FBBBR has been shown in in vitro studies to inhibit the production of NO and pro-inflammatory cytokines compared to unfermented and isolated forms [102].

Another in vitro study isolated the compound from *Scutellaria baicalensis* with greater anti-inflammatory and antioxidant activity, namely wogonin, a ubiquitous polyphenol in vegetables. Wogonin exerts its anti-inflammatory action by upregulating the expression of HO-1 [103].

Finally, a very well-known flavonoid is *quercetin*, found in tea, onions, broccoli, beans, cereals, apples, and grapes, which has already been studied for its anti-allergic properties. In AD models of keratinocytes, quercetin significantly increases the expression of antioxidant factors such as SOD1 and SOD2, CAT, and GPx, to a greater extent than dexamethasone, and counteracts the oxidizing effects of cytokines such as IL-4, IL-13, and TNF-α [104]. The quercetin derivatives, luteolin quercitrin and isoquercitrin, found in Rhodiola crenulata, also have antioxidant effects on the skin. In particular, aryl hydrocarbon receptor (AHR) agonists are hyperexpressed on the surface of keratinocytes. AHR regulates the skin barrier by promoting the expression of the barrier proteins LOR, IVL, and FLG [105].

Flavonoids, and more broadly polyphenols, are powerful natural compounds that exert their bioactive function at the level of various organs and tissues, playing an important role in the regulation of neuronal functions, in learning and memory processes, and in counteracting neuroinflammation.

Many of the flavonoids mentioned above, which are involved in the regulation of oxidative stress in AD, have been shown to play a role in the regulation of psychological disorders such as anxiety and depression. Once hydrolyzed by intestinal enzymes, their bioavailability is highly variable and many can cross the blood–brain barrier and exert neuroprotective effects [106].

Chlorogenic acid has an anxiolytic effect by acting at the GABA-A receptors of benzodiazepines [107]. The role of chlorogenic acid as an antidepressant may be related to the regulation of the opioidergic pathway, but current results are still very controversial [108]. Instead, quercetin has a proven antidepressant effect similar to that of imipramine and fluoxetine, is a weak inhibitor of MAO-A, and has an anti-stress effect by normalizing adrenocorticotropic hormone and cortisol levels following activation of the hypothalamic–pituitary–adrenal axis [109,110].

### 4.2. Alkaloids

Alkaloids are a class of metabolites that are structurally very diverse and complex, but all share the presence of an amino acid as a nitrogen source [111]. They are mostly of plant origin, particularly in the *Leguminoseae* and *Solanaceae* families, but can also be derived from fungi, such as ergot alkaloids [112,113]. Chemically, they are bases, but neutral alkaloids or others containing quaternary ammonium salts have also been found in nature, and the biological function of these types of alkaloids depends precisely on the transformation of the amine function into an ammonium salt [114].

The alkaloids derived from *Dictamnus dasycarpus* Turcz., a member of the Rutaceae family, are dictamine and haplopine, and, together with the limonoids contained in the plant, have shown anti-inflammatory and antioxidant properties in mice with AD and in vitro on human cells.

Haplopine induces an increased expression of SOD and CAT in cells subjected to oxidative stress and it reduces the levels of pro-inflammatory cytokines such as IL-6, GM-CSF, G-CSF, and TSLP in human keratinocytes, and IL-4, IL-13, and COX2 in human T lymphocyte cells. It also appears to reduce mast cell infiltration and serum IgE concentrations in affected skin. Moreover, haplopine appears to reduce IL-6 expression more than that achieved by tacrolimus, all of which suggests its potential role as an anti-AD agent [115].

### 4.3. Terpenes

Terpenes are a large class of natural compounds of animal and plant origin, classified as non-saponifiable lipids. They can be classified according to the number of carbon atoms in their chemical structure as monoterpenes, sesquiterpenes, diterpenes, triterpenes, and their derivatives [116]. They are the main constituents of plant resins and essential oils, and many flavors used in foods or perfumes are derived from natural terpenes or terpenoids. Their primary function is to give the plant, and especially the resin on it, a particular fragrance that distinguishes it from others, but over time, the beneficial functions of these substances for human health have also been discovered [117]. Loganin is a monoterpene found in *Cornus officinalis* which, together with the flavonoid naringenin, has shown anti-atopic effects in an in vitro model of AD. In particular, the synergistic antioxidant function of the compounds contained in the Cornus officinalis extract, including loganin, counteracts the action of ROS and consequently the formation of a pro-inflammatory microenvironment, inhibiting TNF-α-induced apoptosis in HaCaT cells. In addition, in silico findings suggest that these bioactive compounds reduce skin inflammation by preventing the binding of IgE to FceRI [118]. In the area of mood disorders, loganin shows antidepressant effects in mice, acting on the serotonin pathway and increasing levels of 5-hydroxytryptamine in the prefrontal cortex, hippocampus, and striatum [119]. Naringenin is a more potent MAO inhibitor than quercetin, also showing a possible role in the treatment of depressive status [108].

*Arjunolic acid*, found in the plant *Terminalia arjuna* (*Combretaceae*), is a triterpenoid saponin known for its antioxidant properties. It plays a protective role in the heart and in diabetic vasculopathy. It has recently been shown to be beneficial in mice with AD, improving skin thickness, pruritus, and dermatitis score. On the one hand, arjunolic acid inhibits COX, which has an anti-inflammatory effect, and, on the other hand, it improves the oxidative state of skin affected by AD, favoring the scavenging of ROS, and reducing serum IgE levels, TNF-α, and IL-6 [120].

Among the terpenes, the carotenoids have important anti-inflammatory and protective functions in the cardiovascular, gastrointestinal, hepatic, neurodegenerative, and cutaneous fields. In particular, *astaxanthin* is a xanthophyll carotenoid found in microalgae and crustacea such as krill and shrimp, and its liposomal formulation has been shown to be effective in the topical control of AD. The efficacy at the cutaneous level seems to be due to the ability of astaxanthin to restore the balance between oxidizing factors and antioxidant protective factors in AD, since treatment with it could reduce the levels of MDA and H_2_O_2_, while at the same time increasing the glutathione/GSSG ratio and restoring HO-1 and GPx-1 expression. It also reduces TNF-α, IL-1β, and IL-6 levels and serum IgE concentration [121].

The increased dietary intake of carotenoids has been associated with a lower incidence of anxiety, depression, and stress. Instead, a low-carotenoid diet is related to cognitive impairment. Carotenoids could play their neuroprotective role by acting on neurotrophins and MAO and reducing the expression of pro-inflammatory cytokines with IL-6 and TNF [122].

### 4.4. Isothiocyanates

Isothiocyanates, or glucosinolates, are particularly known in the phytotherapeutic field for their lacrimatory, revulsive, rubefacient, and vesicular action.

They are also responsible for the pungent smell and spicy taste of the foods that contain them, and through this mechanism in nature, they discourage the aggression of the plant by insects and herbivores. In humans, they appear to have the same effect on cancer cells, as they inhibit certain stages of carcinogenesis and induce apoptosis of numerous cell lines [123].

*Sulforaphane* is an isothiocyanate belonging to the family of sulfur compounds, which occurs naturally in high concentrations in plants of the cruciferous family (broccoli, cabbage, and Brussels sprouts) [124]. It acts as an antioxidant on the skin exposed to ionizing radiation, which has been demonstrated in the field of cerebral and cutaneous hemorrhages. On the skin affected by AD, sulforaphane seems to improve the oxidative state by increasing Nrf2, a transcription factor that increases the expression of antioxidant genes and detoxifying molecules, including HO-1; Nrf2 explains a protective action against oxidative modifications in keratinocytes, melanocytes, and fibroblasts. These functions are expressed as an improvement in skin thickness, a reduction in IgE levels, and cutaneous inflammation mediated by eosinophils and mast cells. Finally, Nrf2 appears to be involved in restoring the homeostasis of the skin barrier by activating skin-protective proteins, but the details of this mechanism have not yet been identified [125].

Sulforaphane showed antidepressant and anxiolytic effects on acute and chronic stress, with results similar to those obtained with fluoxetine. The neuroprotective effects on depression and anxiety may be due to the regulation of the hypothalamic–pituitary–adrenal axis and the reduction in the inflammatory state by sulforaphane, pathogenetically correlating chronic phlogosis to the genesis of depressive and anxious behaviors [126].

### 4.5. Other Compounds

Other plants and algae have been described in the literature to have beneficial effects on AD. However, for these plants, the bioactive compounds responsible for their nutraceutical action have not yet been defined, despite the mechanisms for regulating oxidative stress being similar to those previously described for other bioactive substances. We report below the main species that have been shown to have an effect on improving the oxidative state of keratinocytes and other skin cells in AD.

*Alnus sibirica* is a tree whose bark is used for various medical purposes, such as antipyretic, expectorant, and antiasthmatic effects. The fermented bark of *Alnus sibirica* showed better antioxidant action on AD than the unfermented form, in terms of the scavenging activity of ROS and the inhibition of NO production [127].

*Soshiho-tang* is an ancient Chinese medicinal herb used in Northeast Asia with various anti-inflammatory, antineoplastic, and immunoregulatory pharmacological activities. In the regulation of oxidative stress, this herb causes an increase in the expression of Nrf2 and therefore of HO-1, thereby enhancing the protective antioxidant mechanisms [128].

*Sophora flavescens* Aiton, a member of the *Leguminosae* family, has long been used in Eastern countries to treat gastrointestinal bleeding, diarrhea, and eczema. Various phytocompounds have been isolated from it, such as quinolizidine alkaloids, flavonoids, triterpenoid saponins, ethanolic extracts, and (2S)-20-methoxykurarinone. Its extract has an antioxidant and anti-atopic effect, mainly through the upregulation of HO-1, although it has not been possible to identify whether one of the above phytocompounds is more responsible for this function or whether, more likely, the different phytocompounds compete in a synergistic way to improve the inflammatory state of atopic skin [129,130].

*Sacran* is a polysaccharide sulphate extracted from the seaweed Aphanothece sacrum, which is consumed in Japan. Sacran has the ability to form a gel-like film in the presence of polyols that are resistant to chemical penetration. The special physical properties of sacrum could be used to create a protective barrier on atopic skin when the sacrum is applied to the skin. However, this polysaccharide may not only have a barrier function but also a therapeutic function by regulating the smear and oxidative state of the skin. Corneocytes observed after Sacran application showed larger dimensions, a lower SS/SH ratio, a lower TEWL, and intracellular ROS formation [131].

Table 2 summarizes the plant species and their main bioactive compounds found in the literature, relating to each of them their beneficial effects in regulating oxidative stress in AD and their potential therapeutic effects on psychological disorders.

**Figure 2:**
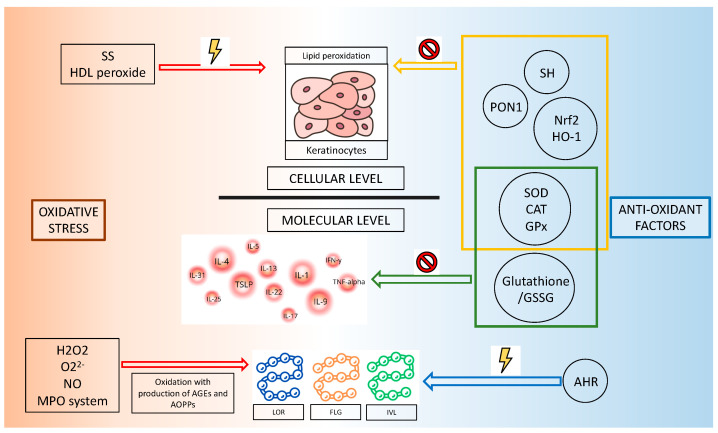
Effects of redox imbalance on keratinocytes, skin barrier proteins, and inflammation mediators (SS = disulfide; SH = thiol; PON1 = paraoxonase 1; Nrf2 = nuclear factor erythroid 2-related factor 2; HO-1 = heme oxygenase 1; SOD = superoxide dismutase; CAT = catalase; GPx = glutathione peroxidase; GSSG = oxidized glutathione; AHR = aryl hydrocarbon receptor; H_2_O_2_ = hydrogen peroxide; O_2_^2−^ = superoxide; NO = nitric oxide; MPO = Myeloperoxidase; AGEs = Advanced glycation end products; AOPPs = Advanced Oxidation Protein Products; FLG = filaggrin; IVL = involucrin; LOR = loricrin).

Figure 2 explains the impact of oxidative stress markers and the role of antioxidant mechanisms on the cellular level (keratinocytes) and on the molecular level (skin barrier proteins and cytokines).

## 5. Conclusions

AD involves a large percentage of the population and it has a significant impact on patients’ quality of life. In addition, patients with AD would appear to have a higher risk of manifesting psychological disorders such as alexithymia, anxiety, and depression, going through social isolation. Furthermore, stress conditions worsen and exacerbate AD symptoms, causing disease flare-ups. Among the pathogenic mechanisms underlying AD, an important role seems to be played by oxidative stress caused by various types of triggers (including psychological stress). Sustained oxidative stress contributes to atopic march and it is one of the driving mechanisms of chronic inflammation, so the employment of antioxidant substances may be useful in reducing inflammatory processes.

Several studies currently reported in the literature highlight a potential role of nutraceuticals (flavonoids, alkaloids, terpenoids, isothiocyanates, and others) in reducing oxidative stress in AD. The pathways mainly involved appear to be a reduced mRNA expression of iNOS and increased scavenging of ROS; an increase in the expression of SOD and CAT and in the SS/SH ratio, counteracting lipid peroxidation and restoring the balance between oxidant and antioxidant factors; a reduction in MDA and H_2_O_2_ by the intervention of glutathione; and an increased Nrf2 gene expression and consequently HO-1, which restore skin barrier homeostasis by exerting antioxidant action.

To date, the main markers of oxidative stress in the pathogenesis of AD have been the generation of ROS and increased expression of iNOS, the role of the MPO system and lipid peroxidation, and finally the role of MDA and the MDA/melatonin ratio. Therefore, the nutraceuticals and bioactive compounds identified in our analysis have a role in regulating most of these oxidative stress mechanisms. However, we did not find any bioactive compounds acting on the MDA/melatonin system.

In addition, AD can often be difficult to treat, so other therapeutic tools need to be evaluated in addition to traditional treatments. A better characterization of patient phenotypes (identifying useful biomarkers and stratifying by degree of disease and the presence of any comorbidities) would be necessary to achieve this goal.

In particular, the use of certain nutraceuticals could help in steroid sparing, reducing adverse effects, and enhancing traditional therapies and could probably also be useful in decreasing psychological stress (e.g., chlorogenic acid, quercetin, naringenin, loganin, carotenoids, sulforaphane). In fact, the latter also exerts neuroprotective effects, reduces the inflammatory state, and regulates the hypothalamic–pituitary–adrenal axis; some of these seem to act as MAO inhibitors, others bind the GABA-A receptors of benzodiazepines, and still others increase the levels of 5-hydroxytryptamine in the prefrontal cortex, hippocampus, and striatum.

Appropriate and timely treatment of this disease can significantly improve patients’ quality of life and limit the development of more severe forms or associated comorbidities.

## Figures and Tables

**Table 1:** Principal oxidative stress markers involved in AD and the corresponding antioxidant factors.

Oxidant Factor	Marker	Effects	Trigger	Antioxidant Factor
ROS (O_2_^2−^, H_2_O_2_, and NO)	iNOS	Damage of dermal microvasculature [20]	Proinflammatory cytokine environment, elevated levels of glucocorticoids	Tocopherol [33], SOD, and CAT [30]
	AOPPs	Oxidation of amino acid residues and loss of biological activity of barrier proteins [23]		
MPO system	HOCl	Production of AGEs, macrophage activation, endothelial damage [18]	Staphylococcus aureus infection	/
	Lipid hydroperoxides	HDL peroxidation and its loss of function [22]	Activated leukocytes	PONs (PON1)
SS	SS/SH ratio	Lipid peroxidation of keratinocytes [24]	/	Serum thiols and glutathione/GSSG
Urinary nitrate	8-OHdG	Predictive value of progression of atopic march, increased risk of asthma onset [27,28]	/	/
	MDA		/	Melatonin [32]

ROS: reactive oxygen species; O_2_^2−^: superoxide; H_2_O_2_: hydrogen peroxide; NO: nitric oxide; iNOS: inducible nitric oxide synthase; AOPPs: Advanced Oxidation Protein Products; SOD = superoxide dismutase; CAT = catalase; MPO: Myeloperoxidase; food: hypochlorite; AGEs: Advanced glycation end products; PONs: paraoxonases; PON1: paraoxonase 1; SS: disulfide; SS/SH ratio: disulfide/thiol ratio; GSSG: oxidized glutathione; 8-OHdG: 8-Hydroxy-2′-deoxyguanosine; MDA: malondialdehyde.

**Table 2:** Main classes of natural compounds, the plant species and possible bioactive compounds identified, and the corresponding effects on AD and psychological disorders.

Natural Compounds	Plants and Fruits	Bioactive Ingredient	Effects on AD	Effects on Psychological Disorders
Flavonoids	Wheatgrass and Aronia melanocarpa	Chlorogenic acid	Reduction in mRNA expression of iNOS, COX2, NO, and PGE2, and increase in antioxidant factors (Nrf2 and HO-1) [98]	Antidepressant action on the GABA-A receptors of benzodiazepines, regulation of opioidergic pathway [107,108]
	Nymphoides peltata	Chlorogenic acid + caffeic acid	Activation of Nrf2/HO-1 [99]	/
	Coffea arabica	Chlorogenic acid + caffeic acid	Reduction in intracellular ROS inhibition of lipid peroxidation, increase in antioxidant enzymes (CAT) [100,101]	/
	Blueberries and black rice	Anthocyanins and derivatives of gallic acid supplemented in Lactobacillus plantarum	Inhibition of NO production [102]	/
	Scutellaria baiacalensis	Wogonin	Upregulation of HO-1 [103]	/
	Tea, onions, broccoli, beans, apples, grapes, Rodiola crenulata	Quercetin, luteolin quercitrin, and isoquercitrin	Increase in SOD1 and SOD2, CAT, GPx, and HO-1, promoting the expression of skin barrier proteins [104,105]	Antidepressant inhibitor of MAO-A, anti-stress effect by normalizing adrenocorticotropic hormone and cortisol levels [109,110]
Alkaloids	Dictamnus dasycarpus Turcz.	Dictamine, haplopine	Reduction in COX2, increase in SOD and CAT expression [115]	/
Terpenes	Cornus officinalis	Loganin	Counteracting the action of ROS [118]	Antidepressant, acting on the serotonin pathway [119]
		Naringenin		MAO inhibitor [108]
	Terminalia arjuna	Arjunolic acid	Inhibition of COX, scavenging of ROS [120]	/
	Microalgae and crustacea	Astaxanthin (carotenoid)	Reduction in MDA and H_2_O_2_ levels, increase in glutathione/GSSG ratio [121]	Antidepressant, anti-stress, and anxiolytic action of carotenoids on neurotrophins and MAO [122]
Isothiocyanates	Cruciferous family	Sulforaphane	Increasing in Nrf2 and HO-1 [125]	Antidepressant and anxiolytic effects by the regulation of the hypothalamic–pituitary–adrenal axis [126]
/	Alnus sibirica	/	Scavenging of ROS, inhibition of NO production [127]	/
/	Soshiho-tang	/	Increase in Nrf2 and HO-1 [128]	/
/	Sophora flavescens Aiton	Synergistic action of quinolizidine alkaloids, flavonoids, triterpenoid saponins, ethanolic extracts, and (2S)-20-methoxykurarinone	Upregulation of HO-1 [129,130]	/
/	Sacran	/	Lower SS/SH ratio and intracellular ROS formation [131]	/

iNOS = inducible nitric oxide synthase; COX2 = cyclooxygenase 2; NO = nitric oxide; PGE2 = prostaglandin E2; Nrf2 = nuclear factor erythroid 2-related factor 2; HO-1 = heme oxygenase 1; ROS = reactive oxygen species; CAT = catalase; SOD1 = superoxide dismutase 1; SOD2 = superoxide dismutase 2; GPx = glutathione peroxidase; MDA = malondialdehyde; H_2_O_2_ = hydrogen peroxide; GSSG = oxidized glutathione; SS = disulfide; SH = thiol.
